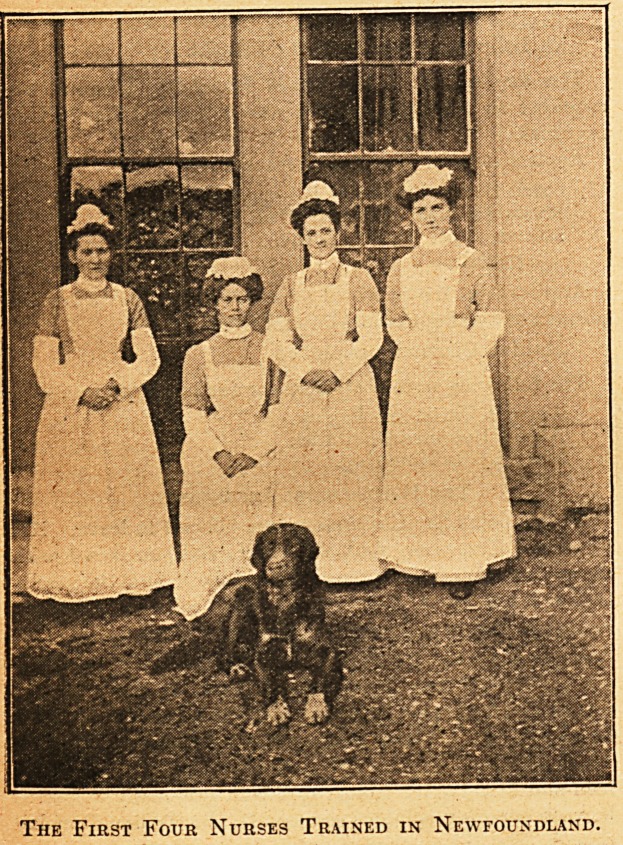# The Hospital. Nursing Section

**Published:** 1907-03-09

**Authors:** 


### The Hospital.

**Nursing Section.**

Contributions for "The Hospital, should be addressed to the Editor, "The Hospital" Nursing Section, 28 & 29 Southampton Street, Strand, London, W.C.

No. 1,069.—Vol. XLI. SATURDAY, MARCH 9, 1907.

### Notes on News from the Nursing World.

### THE QUEEN AT THE JUBILEE INSTITUTE.

On Wednesday afternoon Her Majesty Queen Alexandra paid a surprise visit to the Jubilee Institute for Nurses in Victoria Street. She was received by several members of the Council and the officials, and listened with keen attention to all that was told her. The visit lasted for upwards of a quarter of an hour, and has not only given the utmost satisfaction to the authorities of the Institute, but will excite a great interest throughout the country. There is no doubt that Her Majesty's gracious visit will afford genuine encouragement to many a district nurse whose isolation in her work often constitutes its chief burden.

### ROYAL VISIT TO GUY'S HOSPITAL.

On Thursday afternoon last week the Prince and Princess of Wales visited Guy's Hospital. The visit was informal. They were conducted by Sir Cooper Perry and the matron, Miss Swift, round some of the wards, also to the Light Department and the Medical School. In the wards they chatted with some of the patients and asked various questions concerning the accident cases. They left about 5.30 P.M., and quite a crowd collected in the entrance quadrangle to see them off.

### THE QUEEN AND THE LATE MISS GUTHRIE WRIGHT.

The death of Miss Guthrie Wright, at the age of 62, has deprived the nursing movement in Scotland of one of its pioneers. Miss Guthrie Wright, in her earlier years, displayed keen interest in the extension of education amongst women, and her activity and great breadth of sympathy were brought to bear upon every undertaking with which she was associated. She took a prominent part in the inauguration of the Scottish Branch of Queen Victoria's Jubilee Institute, and acted as hon. secretary from the start in 1887 until the date of her death last week. In fact, the success which the institute has attained over the Border is largely due to her untiring efforts, and she personally raised upwards of £20,000 for the work. Her remains were laid to rest in the Dean Cemetery, Edinburgh, on Thursday, the 28th ultimo, a funeral service being previously conducted in St. Mary's Cathedral by the Bishop of Edinburgh, who also took the service at the grave. There was a notable gathering of mourners, including many persons connected with the Jubilee Institute. The following telegram was received by Miss Louisa Stevenson, who represented the Princess Louise Duchess of Argyll at the funeral: "Queen Alexandra desires me to express her sympathy and sense of the Institute's sad loss sustained by the death of Miss Guthrie Wright." Many other telegrams were sent from distinguished persons, showing the high esteem in which the deceased lady was held in the country, as well as in the city where she lived and laboured for the benefit of all classes of the community.

### WHITECHAPEL INFIRMARY SCHOOL RECOGNISED BY THE MIDWIVES BOARD.

There is an impression among poor-law officials that the Central Midwives Board do not recognise any poor-law institution as a training school. The speaker who at the recent Poor-law Conference showed that he shared this delusion should read the list of names of successful candidates at the February examination of the Midwives Board, which we printed last week. The number of schools at which those candidates were trained included nine poor-law institutions. The Board agreed at their meeting last week to recognise Whitechapel Poor-law Infirmary as a training school; and other poor-law institutions previously approved under Section 6 of the Rules are Fulham, Greenwich, Kensington, Lewisham, Shoreditch, Birmingham, Kings Norton, Croydon, Liverpool (Brownlow Hill and Walton), Nottingham, Sheffield, West Ham, Chorlton, and Cardiff Union Infirmaries—some of the best-known poor-law training schools for nurses in the country.

### NOT EVEN FOURTH CLASS.

When a medical inspector of the Local Government Board makes an unsatisfactory report of the sick wards of a workhouse, and Mr. Preston Thomas advises the Board of Guardians responsible to ask for the resignation of a nurse, the presumption is that the state of affairs is not what it should be. The Chard Guardians, who are now advertising for a nurse, decided at their last meeting, at the instance of the House Committee, to adopt the advice of Mr. Preston Thomas. But two of them expressed an opinion that the nurse, who has been in charge of the sick wards at the Chard Workhouse for nine years, did not deserve blame. The chairman of the House Committee said that the arrangements in the wards are "neither first, second, third, nor even fourth class"; and Dr. Fuller, the medical inspector of the Local Government Board, described the patients as "dirty." It appears to us that if these allegations are accurate, the Guardians themselves are at fault. Why did they allow the arrangements of the infirmary under their control to become not "even fourth class"?

### NURSING ASSOCIATIONS AND BUSINESS PRINCIPLES.

Several serious statements were made by Mrs. Samuel Courtauld at a meeting of the subscribers to the Halstead and District Nursing Association the other day. The report having been read, Mrs. Courtauld complained that no committee meeting had taken place for four years, and that no balance-sheet had been issued during that time. The nurse, she said, visited a class of people she was never intended to serve, and there were no rules to obey. The accuracy of these statements was not disputed, and it was eventually agreed "to organise the Association." But we are not surprised that, in the absence of organisation, subscriptions were refused. It is absolutely essential that nursing associations should be conducted on business principles, that meetings should be periodically held, and reports with balance-sheets properly audited, regularly issued. Unless these elementary conditions are complied with, the public do well to withhold support.

### THE MISTAKE OF A WORKHOUSE MASTER.

The former superintendent nurse at Auckland Workhouse Infirmary, Miss Bowman, last week appeared as plaintiff at the Durham Assizes, in an action for slander against the Chairman of the Shildon Urban District Council. The alleged slander was contained in the statement of the defendant, at a meeting of the Auckland Guardians, that the plaintiff had "worked" up a case of insanity against an aged woman in the wards in order to relieve herself of the trouble of looking after the patient. The statement was not denied, but the defendant, in his evidence, said that his remarks did not apply to the superintendent nurse, and the jury accordingly found in his favour. We do not say that they were wrong, but we observe that in the course of the trial mention was made of the fact that the workhouse master told the superintendent nurse that the guardians had decided to put the patient in question into a smaller room by himself in charge of two of the inmates. The superintendent nurse, it was added, declined to obey the instructions, on the ground that "the regulations forbade the nursing of a pauper patient by the other inmates of the workhouse." It subsequently transpired that the workhouse master had misconstrued the order, which was that "two attendants should look after the woman," but we are glad that Miss Bowman had the courage to refuse to comply with the request as it was conveyed to her. Miss Bowman has now ceased to be superintendent nurse, and it would be better for the Auckland Guardians to give their instructions to her successor direct, and not through the medium of the workhouse master.

### A CASE WITH A MORAL.

An action tried at the recent Hampshire Assizes at Winchester in which the plaintiff was Mrs. Firminger and the defendant Mr. B. J. Hewitt, a gentleman of independent means residing at Bishop's Waltham, merits attention because the plaintiff described herself as a nurse. It appears from the evidence that in July last year she answered an advertisement in our columns for a nurse-companion representing herself as "hospital trained." She subsequently definitely stated that she was trained at the West Ham Hospital. She obtained a situation, but after being in the employ of Mr. Hewitt for a month was dismissed because she failed to give satisfaction. As soon as she left she made charges of indecent conduct against the defendant, who is totally blind and paralysed, and then brought proceedings against him with the view of obtaining compensation for wrongful dismissal. Inquiries being instituted respecting her antecedents, it was ascertained that the only training she received was as a pupil midwife for two months in 1900 at Queen Charlotte's Hospital, and for three months as probationer at West Ham Infirmary. We are not concerned with other details of her career, but the fact that when the case came before the court she refused to go into the witness-box proved both that her charges against Mr. Hewitt were absolutely unfounded, and that she did not care to undergo the ordeal of cross-examination. We think it is a pity that when she stated, before her engagement, that she was trained at West Ham Hospital, an authoritative confirmation of her statement was not sought. An official denial would have precluded her engagement, and saved Mr. Hewitt all the annoyance and trouble to which he was subsequently subjected.

### A DELICATE QUESTION AT BATH.

Complaints having been made that when the authorities of the Bath District Nursing Home sends out nurses for confinement cases they draw a distinction between legitimate and illegitimate births, Mr. Plowman, at the last meeting of the Sanitary Committee of the Corporation, explained the position. The governing body of the Nursing Home in Rivers Street are the administrators of an old Bath charity called the Puerperal Charity, and under the conditions of the trust the cases attended must be necessitous respectable married women. Therefore, he added, it is not possible for the institution to send the nurses to illegitimate cases. Mr. Plowman also added that nurses are sent out to patients who pay a small fee; but that as a rule illegitimate cases are not taken "because the institution cannot deal with all the legitimate births; moreover, their nurses can only look after patients as a rule for ten or twelve days, and in such cases the nurses run considerable risk, and might have to be in quarantine, which would be disastrous for their regular work." He believed that as the workhouse keeps the mothers a month it is better that they should go there. The question whether district nurses provided by public subscription should be allowed to attend illegitimate births is one constantly recurring. In our opinion these are unquestionably Poor-law cases, and this for more than one reason. The unmarried mothers commonly need more substantial help than can be supplied by the nurse, and in the workhouse they are well cared for and severed from gossiping friends.

### AN EXPERIMENT FOR TWELVE MONTHS.

It has just been decided by the King's Norton Guardians to authorise the medical officers in the area under their control—which includes parts of the city of Birmingham—to accept directions from a registered midwife to call upon a woman in circumstances of difficulty or danger, subject to the stipulation that the midwife shall only use such a call when there is a necessity for immediate medical help, and when she is satisfied that there is inability to pay for ordinary medical help. This arrangement, which is to be tried for a year as an experiment, has been determined upon in order to avoid any risk of women losing their lives through lack of medical assistance.

### THE CARNIVAL THAT FAILED.

At the last meeting of the Hammersmith and Fulham Nursing Association two notable announcements were made. One had reference to the future and the other to the past. The general meeting will take place on the 13th of May at Fulham Palace, the Bishop of London presiding. This should help to make up for the disappointment caused by the announcement that the sum of money divisible between the association and two other philanthropic societies from the proceeds of the Hammersmith Regatta and Carnival of last year was only £14 18s. 2d. This result occasioned all the more surprise because the surplus of the preceding carnival was £156 0s. 7d. The difference is startling, and it is emphasised by the fact that the gross receipts on the first occasion were £358 18s. 3d., and last year £217 15s. 10d. The expenditure to secure £15 was only a few pounds less than that incurred to obtain upwards of £150. In these circumstances and as the receipts last year included subscriptions £75 3s. 6d., the sooner an entertainment not so costly to organise is substituted for the regatta, the better for the Hammersmith and Fulham Nursing Association.

### THE SALARIES OF MENTAL MALE ATTENDANTS.

It is satisfactory to learn that, at the instance of the Asylum Committee of the London County Council, the male attendants in the institutions under the Council are to receive an increase of pay, and that in future there will be no deductions from salaries for misconduct. Offences are to be dealt with, as they should be, by other means. A statement was made the other day by Dr. Hogg at Hanwell Asylum, which tends to show that the standard among these attendants is steadily improving. The coroner, at the conclusion of an inquest on an inmate—who died from exhaustion following a desperate struggle with an attendant which the patient had provoked, the attendant barely escaping with his life—said that in 15 years he had only had to attend four inquests at Hanwell. Bearing in mind that there are more than 2,600 patients in the asylum, he considered that fact remarkable, and we think it will be generally agreed that it speaks well for all the officials.

### QUEEN'S NURSES IN EAST LONDON.

The annual meeting of the Shoreditch and Bethnal Green District Nursing Association was held on Monday evening. A feature of the report is the references to the changes in the executive. The Vice-Chairman, the Rev. H. V. S. Eck, has become chairman, and Miss Peter, formerly general superintendent of the Queen's Jubilee Institute, is now hon. secretary. There is no doubt that the great experience which Miss Peter has had of nursing work will be employed to the advantage of the organisation. Mention is also made in the report of the death of Miss L. D. Sparkes, who for eighteen years acted as hon. treasurer. As to the record of the past twelve months, there were no fewer than 1,922 cases nursed, and the total number of visits paid was 42,520. It is interesting to learn that 857 of the cases were sent in by medical men, 487 by the clergy and church-workers, 239 by the friends of patients, 119 by hospital authorities, and 55 by sisters of St. Saviour's Priory, while 30 were discovered by the nurses. Of the patients during the period 1,333 are now convalescent, 231 were transferred to hospital or infirmary, and 215 died. The financial position might be worse because there is a balance of £27 4s. 2d. on the side of income, but it might be better, for the work is increasing and more money in subscriptions is needed before an addition can be made to the staff.

### NEW HOME FOR LEICESTER INFIRMARY NURSES.

At an estimated cost of £20,000 the Governors of Leicester Infirmary propose building a nurses' home for the nurses connected with that institution. Some three or four years ago the accommodation for nurses at the infirmary was so limited that it was found necessary to lease a house on the outskirts of the town. The administration, however, of such a large house away from the institution itself has resulted in a very considerable annual expenditure, and this fact and the knowledge that the new wing of the infirmary will necessitate a substantial addition to the nursing staff made the provision of adequate and proper accommodation for the latter a matter of urgent necessity.

### NON-TRAINED NURSES AND THE MIDWIVES ACT.

It is worthy of note that all the four pupils trained by the Cardiff Queen's Nurses' Institute Maternity Department passed the recent examination of the Central Midwives Board, and that three were not trained nurses. This should have the effect of encouraging eligible young women who wish to take up the work of a midwife, and have not been able to undergo the training of a nurse, to endeavour to qualify under the Act.

### SHORT ITEMS.

The Secretary of the Royal National Pension Fund for Nurses desires to acknowledge through our columns a postal order for £1 which has been sent to him anonymously as a contribution to the Benevolent Fund.—The successful candidates at the recent examination of the Central Midwives Board include Nurse A. M. E. Molony, who was trained at the County and City of Cork Lying-in Hospital.—The members of the Stockton and Thornaby District Nursing Association had the pleasure of a visit on Thursday last from Miss Amy Hughes, general superintendent of the Queen's Jubilee Institute, who gave an interesting address to the House Committee.—At the next meeting of the Medico-Legal Society, which will be held on Tuesday evening, March 12, at 8.15, in the rooms of the Royal Asiatic Society, 22 Albemarle Street, W., Mr. J. Theodore Dodd, M.A., will introduce a discussion on the working of the Midwives Act, and several members of the Central Midwives Board have promised to attend.—A kindly reference was made at the annual meeting of the Governors of the Buchanan Hospital, Hastings, to the death, in June last, of Miss Ransford, who for fourteen years acted as matron.

### The Nursing Outlook.

"From magnanimity, all fears above;
From nobler recompense, above applause,
Which owes to man's short outlook all its charm."

### MAKE-BELIEVE IN BRITISH NURSING.

### IV. HOW FICTION WAS DRESSED AS FACT.

THOSE of our readers who have read our three previous articles on the Stage Army, its methods, its leaders, and the way in which the Matrons' Council has been made to do service for the National Council of Nurses, the International Council of Nurses, and the Society for State Registration, may have wondered how the promoters have contrived to make these paper houses look so real? It may be assumed that these manœuvres might be taken seriously in the United States, although the British nursing world has recognised them, from the first, as nothing but the same Stage Army, dressed to suit each new part assigned to it, in the constantly changing scene. The six or eight moving spirits who have formed the skeleton of the Stage Army are entitled to plume themselves on the temerity and ingenuity with which they have organised the numerous masquerades. The manœuvres of the Stage Army have often been intensely funny. So amusing, indeed, have they become, that had it not been for the interests of the profession, we should have hesitated to prick so many coloured and brilliant a bubble. Could anything be funnier than the specimens we gave last week of the literary efforts of the Mutual Admiration Society? But we must pass on to explain how fictions have come to be dressed like facts.

At one time, at any rate, there was reason to believe that in America the Stage Army, the leaders of which have christened themselves the Matrons' Council, the International Council of Nurses, the Society for the State Registration of Nurses, and the National Council of Nurses, may have been taken seriously. But in the United States the nursing world, as a whole, is better organised than in probably any other country. There is, for instance, the American Society of Superintendents of Training Schools, which embraces the whole of the leaders of the nursing profession, who are mainly responsible for the education and training of nurses throughout the United States. Each training school has its own *alumnæ* which include the whole of the graduates of the school. These *alumnæ* have been united by the formation of the Nurses' Associated Alumnæ of the United States. These two organisations practically control nursing affairs and fully represent nursing opinion in the United States. They have worked most zealously in promoting the registration of nurses in the States of the Union, and to their efforts much of the improved nursing and the enhanced position of nurses in America is due. In 1900 the American Society of Superintendents, or some of its more active members, conceived and established the *American Journal of Nursing*, which has been ably conducted and is a most interesting and well-written magazine. It follows that nursing matters in the United States are on the whole better organised than elsewhere owing to the unity of feeling and purpose from the outset which has characterised the public proceedings of the superintendents and nurses of the principal nurse training schools. No surprise could therefore be felt, had the leaders of nursing in the United States, or some of them, been led, in the absence of definite information to the contrary, to treat the Matrons' Council, and the councils and societies which constitute its children, as authoritative and influential bodies, representing British nursing in the same way that American nursing is represented by the superintendents and nurses' societies in the United States.

It is satisfactory to note, however, that, with their usual acuteness, the leaders of nursing in the United States appear to have grasped the true inwardness of the facts which we have set forth in this and preceding articles. They seem to have realised that the so-called International Council of Nurses had no representative character, and that it did not include either the leaders of British nursing or the leaders of nursing in other countries. This they felt it must do, before it can justify its title and its proceedings can carry any weight or authority. We have formed this conclusion from an official announcement, circulated throughout the United States, to the effect that the Conference of the International Council of Nurses, to be held in Paris next June, "will be informal." "It is not," therefore, "necessary for associations to send formally accredited delegates," but "nurses will be welcomed and it is hoped that a large number may present themselves." In other words, any nurse who can afford the trip is invited to spend her summer holiday in Paris, in June, to provide her own quarters, and to keep her eye on certain papers, where in due course she will learn "the names of the hotels where the officers of the 'so-called' Council may be found." "There will be no special rates nor official headquarters."

It will be seen that this official notice nicely pricks the bubble of the International Council of Nurses. The Conference is thus shown to be merely a summer's picnic, and all who take an interest in nursing can heartily wish those nurses, who are able to afford the excursion, a pleasant visit to Paris, and full enjoyment of the many facilities that city affords for pastime and amusement.

Finally, it may be usefully remembered that Mrs. Bedford Fenwick, on her return from America, renamed her paper *The British Journal of Nursing* in July, 1902, and that it was afterwards advertised as the "Official Organ" of the Matrons' Council and of the other councils and societies which we have shown to be its children. She further tried to organise nurses' leagues in place of *alumnæ* and a National Council to affiliate these leagues in lieu of the Nurses' Associated Alumnæ of the United States. Our American cousins may reflect that, sometimes, but not always, imitation is the sincerest flattery.

### Nursing in Tropical Climates.

By Andrew Duncan, M.D., B.S., M.R.C.P., F.R.C.S., Fellow King's College, Lecturer on Tropical Medicine at the London School of Tropical Medicine, and the Westminster Hospital.

### VIII. DYSENTERY.

(*Continued from page* 307.)

### Clinical Signs of Dysentery.

The definition already given by Osler embrace the salient points of a case of dysentery in its acute and chronic forms. It may, however, be pointed out that when a patient who is suffering from a very acute form of the disease rather suddenly loses all pain, the observer must not think from this alone that he is beginning to progress favourably, for this sudden cessation of pain may indicate that gangrene of the intestine has set in, and a fatal termination is imminent.

Before proceeding to the actual details of nursing, the consideration of the stools will first be taken in hand.

### Characters of the Stools.

(*a*) The simplest form of stool consists of about a tablespoonful of blood-stained mucus, with a few small clots, and some rounded scybalous matter, surrounded by sanious fluid, or

(*b*) In the milder form of the disease first a solid motion may be passed, covered more or less with greyish or colourless mucus. Next small quantities of offensive mucus with minute fæcal lumps. Then there are passed mainly small quantities of mucus, stained with fæcal matter, and often mixed with blood. And, in addition, scybala are passed occasionally.

(*c*) In the severer forms, after the bowel has discharged its contents, which are often solid, we find whitish or coloured jelly-like mucus quickly becoming bloody, with often pretty large quantities of clotted blood. The motions are frequent and of about two to three drachms in quantity. This stool forms a slightly yellowish, glairy, quivering mass lying in balls or clumps, without any fæces, or else around a formed mass of fæces. If the discharge be very fluid, the masses of mucus unfold into hyaline shreds—the so-called *shreddy stool.* This type of stool is passed in the first stage of the disease.

(*d*) In a more advanced stage there will be a small amount of yellow fluid, generally without fæces, and containing floating in it a number of reddish lumps, as large as a pea or bean, like raw meat. This stool has been named the *Lotura carnea.*

(*e*) The stool may be *purely bloody*, resulting either from superficial ulceration or from ulceration into a large vessel.

(*f*) In the later stages of the disease we may find a *purulent stool*, due to the formation of submucous abscesses or to destruction of the mucous membrane. The pus is either pure and odourless, or, more frequently, is mixed with fæcal matter and blood.

(*g*) *The so-called frog's spawn or boiled sago stool.* This, although occurring in the acute, yet is mainly seen in the chronic varieties of the affection. It consists of clumps of large hyaline mucus. It is supposed to arise by mucus being pressed into the cavities out of which the intestinal follicles have fallen; this mucus is then modelled in their cartil, and again falls out into the bowel.

(*h*) Sometimes we find with the ordinary stool of dysentery that from time to time the patient will pass *fluid fæcal matter* throughout the course of the illness.

(*i*) *Green stools.*—In certain cases these are passed. Buchanan, who has drawn attention to this variety, thinks the colour is due to unchanged pigment in the stools, due to the ulceration in the intestine hastening the peristaltic action, and so causing a quicker passage.

(*j*) *Pulpy stools* have been described by Sir Joseph Frayrer. They are very offensive without blood or mucus, and occur in the gangrenous form of the disease.

(*k*) *The gangrenous stool* has a horribly penetrating odour. It consists of a blackish fluid, containing pieces of gangrenous tissue, and sometimes tubular structures, held by some to consist of separated portion of the intestinal canal, but by Heubner of mucus only.

(*l*) *The stools of amœbic dysentery.*—These, which personally I have never seen, are said to be mucoid, with purulent, greyish, shreddy material; greenish like spinach, or pultaceous, with occasionally large necrobic masses; or dark brownish and liquid, floating in which are greyish white masses of the size of a pin's head, embedded in blood-stained mucus. The amœbæ are best demonstrated in the little whitish masses. The earliest stools are small in amount. The odour is mawkish and not offensive. As the disease advances they become more copious, watery, and less homogeneous, with less blood, and much shreddy material. In the periods of intermission, which occur in this form of dysentery, the stools become pasty and even formed, but mucus is still found either mixed with the fæces or adhering to their surface. It only disappears finally after the patient has passed fully formed fæces for some time.

(*m*) *The stools of chronic dysentery.*—These vary much in character. They are thin, watery, of varying colour, and may be very offensive, with pus and blood mixed in different proportions. Sometimes the blood is so intimately mixed that the whole stool is of a dark brownish colour.

Speaking generally, dysenteric stools are marked by their offensive odour, especially when there is any sloughing process going on in the bowel.

*Method of washing Dysenteric Stools.*—The examination of the stool in dysentery is of equal, if not greater, importance as is that of the examination of the sputum in phthisis, for although in some cases simple inspection of the excreta is sufficient, yet in the great majority of cases, as Macleod points out, the pathological products are mixed up with the feculence in such a way as to conceal them and render it necessary for them to be separated. For this purpose the process of washing dysenteric stools, habitually employed and first introduced by Professor E. Goodeve, at the Calcutta General Hospital, is an excellent one, and is carried out as follows: The stool is received into a vessel of considerable size—*e.g.* the pan of a commode. This is then filled with water poured into it from the height of a foot from a jug or tap. Any masses are then broken up by a glass rod or stick. You allow the material to settle for a few minutes, and then slowly decant the fluid into another vessel, so as to present to view a thin layer. The feculence floats and passes over with the fluid; the pathological products and heavy particles of the feculence subside. What passes out is noted, and then you wash again and again what has remained behind by adding fresh water and decanting until the material is freed of all offensive stuff, when it is transferred to a white plate for examination. This process is generally necessary for any accurate study of the stools in dysentery.

Now this preparation of the stool for the inspection of the physician is important, as by it he is enabled to judge of the amount of blood passed, of the stage of the disease, and whether it is tending towards recovery or not.

(*To be continued.*)

### The Nurses' Clinic.

### THE DISTRICT NURSE AND DISCHARGED HOSPITAL PATIENTS.

As ward sister in a London hospital I often wondered what became of some of our patients when, after many weeks of careful but resultless treatment, it was gently and persuasively suggested to them that they "would feel happier and more comfortable at home." As a Queen's nurse one of my earliest experiences was of finding that a dropsical, paralysed and speechless woman, with the most terrible bed-sores conceivable, had been suddenly returned to a home where not a single preparation had been made to receive her, and where the only person in the house was the very rough and brutal husband to whose ill-treatment the original seizure was commonly ascribed.

Although such a combination of painful circumstances could not often arise, it stamped upon my mind the necessity for communication between the hospital and the district nurse. A post card from the matron would have enabled me to make proper arrangements before the patient arrived, and would have spared the poor creature the nervous terror of being left alone with a man who, even if he had done his utmost, was a clumsy labourer totally unfit to wait upon a woman imperatively needing the most skilled surgical nursing. I found her lying on an ordinary double bed, sagging down in the centre from long use, and it was nearly a week before I could succeed in obtaining a water-bed, collect the various other necessaries, and engage a suitable woman to take care of her between my two daily visits and sleep in the adjoining room.

The district nurse should not only be placed in communication with dying patients, but with that far more numerous class dismissed from hospital at an early stage of convalescence because regarded as plain, straightforward cases that "any idiot" could deal with. I cannot gauge the strangely varying powers of idiots, but I know something of the wonderful capacity of mothers and aunts to complicate the most simple illnesses and retard recovery indefinitely, more especially if they are conscientious and have a zeal "not according to knowledge," or a mind peculiarly open to receive neighbourly—and conflicting—advice. These normal cases, bound to end in cure if even moderately favourable conditions can be maintained, have a still greater claim on the nurse, if a less poignant interest.

Another class of sufferers for whom it would be well to enlist the district nurse's services are those persons discharged from asylums ostensibly "cured," but in reality well known to be subject to the possibility of fresh attacks. The nurse has not, as a rule, received any formal training in mental work, but she is nevertheless a keener and more practised observer than the relatives, and far more likely to perceive the symptoms of returning mania. In addition, she can do much for the improvement of the patient's general health and of his environment, and give advice as to suitable occupation, while her visits afford to the friends, who often spend months of nervous apprehension and almost intolerable anxiety, a moral support which is by no means negligible. If the nurse finds the patient living in circumstances of worry, noise, over-excitement, want of air, she should confer with the doctor and the relatives, and see if it is not possible to move him into surroundings of a more healthy and cheering nature. If he is entirely unfit for ordinary work, she must remember the terrible dullness caused by lack of employment and the disastrous manner in which it acts and reacts upon the mind, and try and suggest some interesting and profitable handicraft. If there is a garden, however small, there can be no healthier exercise than in looking after it, and the amusement has the advantage of costing little or nothing and interesting the patient without exciting him.

Other patients are temporarily dismissed from hospital to wait until a favourable moment arrives to undergo an operation, and the nurse can, of course, exercise a general supervision over these cases, try to establish wholesome conditions of life, build up their strength a little before the ordeal comes, and take care that the sufferers are not overlooked and allowed to wait too long.

There are also among the respectable poor very many sadly complicated cases of chronic illness; the labour and expense of caring for the invalid is heavy, and at times, in spite of the assistance given by her long and frequent visits, the nurse finds the friends on the verge of breaking down. In such circumstances the nearest local hospital, if a bed can possibly be spared, should be induced to take charge of the case, although known to be incurable, for a few weeks or months. This gives the relatives time to recover the fatigue and expense, and the sufferer on returning home, perhaps slightly improved in health, is received with fresh stores of zeal and tenderness. When it can be managed, these periodical visits to the hospital are the salvation of many sorely tried house-mothers who for months at a stretch have never known what it is to get an unbroken night's rest or "a good day's cleanin'."

As suggested before, the district nurse should keep the patients of the Orthopædic Hospital in view, see that the supports continue to fit, are in good order, properly applied, and regularly worn. Doctors have sometimes told me, "I am weary of getting these appliances for my child patients. The little beggars howl, the mothers give in and take off 'the 'orrid nasty things,' and all my time and trouble is wasted," Spectacles come under the same heading; many children refuse to wear the glasses provided, or wear them carefully squinting over or under the rims; while, on the

THE NURSES' CLINIC.—*Continued.*

other hand it frequently happens that the glasses are conscientiously used long after the need for them has passed away, or when the eyesight has changed so radically that their action is positively injurious.

However busy the nurse may be with her own immediate duties, she must always try and spare time for the supervision and organisation without which so much worthy effort is wasted or turned to bad account.

### Incident in an Infirmary Nurse's Life.

OUR SISTER.

"A new patient, sister."

"Not another child, I hope?" said sister, smiling faintly.

"Yes; a baby—three," I answered, not smiling at all. Work was more than usually heavy just then, and all the children of the "House," and most of those outside it, seemed to be packed into the infirmary ailing something, and to me the advent of this last was just the final straw.

"Where shall I put him, please?"

"I will come up with you and see what can be done." The wards were full, and I knew even sister, with all her resource, would be puzzled where to put the new arrival. Many people called our sister hard and most of us thought her cold. Maybe we give it another name now.

At the end of the corridor—such a pretty glass corridor, with hanging baskets and plants of red and white geranium—were the two women who had brought the boy, and, seeing us, one of them—his mother—said: "It's only Sammy, sister."

"I'm afraid I haven't the pleasure of 'Sammy's' acquaintance, but let me have a look at him. Come, Sammy."

A small flaxen head raised itself, and two bare pale arms held themselves feebly out.

"The wee lamb," murmured his mother, "how he goes to her!"

But sister only said "I will bath him, nurse," and to the women, "Visiting day on Sunday."

"They say she's 'ard," I heard one of them say, as they found their way out.

"Aye; but she wain't be hard to Sammy," was the reply—to my thinking a tribute to Sammy for his childish power; to herself for her belief in his power; and to sister, toc, that she would yield to it. I paused a second on my way up the ward to look at Sammy's face.

"Isn't he like a fairy boy, nurse?"

And, indeed, he was. A lovelier child I have never seen. Too white the skin, perhaps, and all too blue the tiny veins; but oh! the sweetness of him. "Congenital valvular disease of the heart" I heard the doctor say in the morning. "He cannot live to grow up, but with care ——" He shrugged his shoulders—"Ah, well, it's from day to day, that is all."

And from day to day sister made it. Her first word was Sammy's and her last service for him. But everybody loved him. His beauty, his infinite patience, and his weakness made him almost the idol of his ward. Was Sammy worse? Harsh voices would soften into whispers. Did Sammy sleep? then every tongue was hushed. Never speaking, sometimes propped high up with a smile of sweet content, sometimes lying with a pinched look of pain, he made his little wants known in a way as wonderful as it was pathetic. Sometimes he would hold his tiny hands as though they hurt him till I sponged them and his face.

But it was to sister in his little of peace, and much of pain, that Sammy used to turn. Sometimes he would languidly put out his hand, and as I passed I would stop to touch it; but he would gently draw it back and feebly shake his head. It was sister he wanted, and she never failed him. To her the tired arms would always stretch, and often she would carry him on a pillow into the sunshine of the garden or the fresh stillness of her own pretty room. It was the morning of a Sunday—visiting day—and sister's long day off duty, when she came up to me with the baby in her arms.

"Sammy seems less well than usual, nurse, and I am taking him down with me. The night nurse reports that he has not slept, and I will have him out a little."

I went to sister's room at dinner-time to see if I should bring him to the ward, and, going in softly lest I should disturb him, I found sister kneeling by his side, stroking his temples with little soft touches in a way he loved. His eyes were closed, and I thought he seemed asleep, but sister, looking up, said, "No, nurse, he's quite awake, but I think perhaps if you give him his warm feed now he might sleep after all. I'll bring him up."

"Do you think he is worse, sister?"

"Yes, he is worse to-day, and if his mother should come up this afternoon ask her to be very still, and if he's sleeping don't allow him to be disturbed for the world. I will see him again after a while."

Sister had been up all night. We had no resident, and I thought she looked very tired and worn. I should like to have fussed over her a little, but she wasn't that kind. There are some people who can't take kindness from anyone, and there are some who can only take it from those who love them. I fancied sister might be one of these.

After his bread-and-milk Sammy did sleep. The pinched look half slipped away, and I thought he had never seemed so fair. Things were very quiet, and the ward had the Sunday peacefulness which we all felt, though I had a shrewd suspicion that the new probationer in the bathroom was mingling her tears with the water with which she scrubbed her mackintoshes. She was very home-sick, poor girl, and it is hard at first to scrub mackintoshes on a hot Sunday afternoon. By and by nothing matters but that they are clean and out of the way. Presently she would appear and take my place at the little table to receive the offerings of affectionate friends, who, perhaps because of the heat, were fewer than usual.

Just then a sound across the ward attracted my attention, and, turning, I was only in time to persuade a pneumonia—perhaps a little forcibly—that he was better on the bed than on the floor. I was firm, and he was expostulating, when a cry rang out that made my heart stand still. It could not have been a loud one, for it was Sammy's, but, oh! the meaning of it. In that one evil moment his mother had come in, startled her boy out of his sleep, and at his cry snatched him in her arms. So natural, so human, and—so fatal!

"You're killing him, can't you see?" I cried, springing over to her at a bound, and gently enough she laid him down. But it was too late. The little blue face told its tale.

I have a confused remembrance of the mother's wailing cry for her bairn, the quick screening of the bed by the probationer, and of a something which, with incredible swiftness, had grasped the child; and was now gently pressing down, now gently stretching up the limp white arms. It was sister, but she who had fought Death for Sammy so often was vanquished now. Just a faint, quivering sigh or two and the little form was still.

With her own hands she folded with fleecy wool the waxen limbs and bore him in her arms away for the last time.

. . . . . . . . .

She had gone again with flowers to heap over him, when his mother came and begged to be allowed to see him too. I took her to the little mortuary, where her loud sobs made sister turn. Taking the mother's hand in hers she said, "Hush! I loved him too, but neither of us would have him back now, if we could, would we?"

My memory went back to the old tribute, for the quieted mother raised her eyes to sister's face and said, "They say you're 'ard, but you ain't bin 'ard to Sammy." And sister—I think sister was just the same: always calm, mostly grave, and sometimes a little stern even. And if we more often saw her still face soften and thought her smile less rare, it was only because we looked to see and knew her better. That was all.

### Meeting of the Central Midwives Board.

The Central Midwives Board met on Thursday, last week, Dr. Champneys in the chair. There were present Miss Wilson, Miss Paget, Sir William Sinclair, Mr. Fordham, Mr. Parker Young, and Mrs. Latter. The Board sat "in camera" for an hour and a half, after which the Press was admitted. The first two items on the agenda were the minutes of the last meeting and correspondence announcing (1) the re-election of Dr. Champneys to represent the Royal College of Physicians on the Board; (2) the re-election of Mr. Parker Young to represent the Society of Apothecaries; (3) the extension of the period for which the present rules of the Board are in force to March 31. These had been disposed of, and the secretary's report of the February examination was then dealt with. Grouping together all the London training schools, the total number of candidates was 145, out of which 116 passed and 29 failed. The greatest number of candidates were sent up by the General Lying-in Hospital and the Plaistow Maternity Charity; but Queen Charlotte's bears the palm in the lowest percentage of failures—excepting those who only sent up one candidate. The total number of candidates from the provincial hospitals was 103, of which 77 passed and 25 failed. Taking into account the candidates from Wales, Scotland, and Ireland, and those who had had private tuition, the total number entered for examination was 389. Out of these 291 were successful, making a percentage of 25.2 failures. The secretary drew attention to the fact that some of the candidates, having done very good papers, were plucked at the *viva voce* through their desire to administer drugs to their patients.

### Poor-law Midwives.

The report of the Standing Committee, which met on February 21, dealt with a letter from Dr. Wheatley, County Medical Officer for Salop, as to the necessity of workhouse nurses holding the Board's certificate. The recommendation was carried that Dr. Wheatley be informed that the position of midwives in poor-law institutions is at present under the consideration of the Privy Council and the Local Government Board. It was also agreed that the same answer be returned to Dr. Niven, Medical Officer of Health for Manchester, who had made inquiries as to notification of intention to practise by a midwife in the Chorlton Union Hospital.

The Committee had under consideration a leaflet—which was produced—distributed to candidates at the recent examination advertising the appearance in a weekly print of February 16 of the correct answers to the questions set. The answers were found to be signed by a medical practitioner approved by the Board as a teacher. The recommendation of the Committee was adopted that the medical practitioner should be informed that the "correct answers" should not be published by an approved teacher until after the oral examination.

### Amendment of Rules.

Certain suggestions had been received from medical officers for the amendment of the revised rules, and it was agreed: (1) That Rule E 5 be amended by the omission in lines 10 and 11 of the words "and then exposed freely to the open air for several days."

2. That Rule E 16 be amended by transposing the last paragraph so as to make it the second.

3. That Rule E 21 (*c*) be amended by requiring the following particulars to be inserted in the notification of stillbirth:—

Full term of premature (number of months) ..................
Condition of child (whether macerated or not) ............
Presentation ..................................................

The revision of the lists of training schools, approved midwives, etc., was referred to the Standing Committee. The following were appointed examiners for the London centre:—

Stanley Dodd, M.B.
G. Bellingham Smith, M.B., F.R.C.S.

The application of the Leeds Maternity Hospital for approval as a training school was postponed for further inquiries.

Applications for approval as teacher:—

Sarah Louise Fraser, M.B.—approved.
Moses Feldman M.R.C.S., L.R.C.P.—refused.
Marion Ethel Unwin, L.R.C.P. and S.L.F.P.S.—postponed.

Applications for approval as a certified midwife for the purpose of signing Forms III. and IV.:—

Matilda Clarkson—approved.

The following were postponed for further inquiries:—

Augusta Christiana Bayley.
Angela Rosamund Griffiths.
Mary Lydia Houlding.

### The Whitechapel Infirmary.

At this point Dr. Champneys was obliged to leave. Mr. Fordham, Treasurer, took the chair, and proceeded with the motion against his name on the agenda "That the resolution of the Board of February 22, 1906, refusing the application of the Whitechapel Infirmary for approval as an institution for the training of midwives, on the ground that the maternity department was structurally unfit for the purposes of a training school, be rescinded, and that approval be now given." There was much discussion on the points which were said to render the building "unfit." Mr. Fordham and Mr. Parker Young had both personally inspected it, with opposing opinions as a result. Sir William Sinclair thought that the number of pupils trained in the institution there in the year did not justify its approval; but with these exceptions the opinion of the Board, based on the fact of the excellent training given by the infirmary, was in favour of the motion, which was accordingly carried.

Whereupon Mr. Parker Young moved: "That the resolution of the Board of November 23, 1905, refusing the application of the Paddington Workhouse for approval as an institution for the training of midwives, on the grounds that the maternity department was structurally unfit for the purposes of a training school, be rescinded, and that approval be now given." As this motion was not seconded it could not be put.

A discussion ensued on certain points in the amendment of the Midwives Act, and Mr. Fordham moved: "That a sub-committee be appointed to consider the matter," which was ultimately carried.

The next meeting of the Board was fixed for March 21.

### General Hospital, St. John's, Newfoundland.

PRESENTATION OF CERTIFICATES.

It is now three and a half years since the first effort was made to establish a training school in the General Hospital, St. John's, Newfoundland. There was a large number of applicants, most of them entirely unsuitable, and of those taken on trial many proved, for one reason or another, unfit for the work. There is still room for improvement everywhere; but, on looking back over the last three years, the results are felt to be decidedly encouraging. The final examination was held in November. Eight nurses entered, and all passed, but only four received certificates, as the other four have some months of their course to finish. The names of the former are Lizzie Redmond, Lizzie Blackmore, Jessie Swyers, and Madge Cullian.

On December 27 a tea and entertainment were given by the ladies of the Cowan Mission. Tea was served in the different wards, and the entertainment was held in the

The First Four Nurses Trained in Newfoundland.

Men's Surgical Ward, which was elaborately decorated for the occasion. Each patient and member of the staff received a gift, and the greater number were well enough to do full justice to the good things provided for tea.

During the evening the certificates and prizes were presented to the winners by the medical superintendent. The prize for first place in the medical nursing examination was gained by Nurse Redmond, who was at work in the hospital some time before the training school was started, and is now night superintendent. The prize for first place in the surgical nursing was gained by Nurse Swyers.

The hospital is quite full, while there are numbers of persons seeking admission who cannot be accommodated. The winter is mild, and consequently unhealthy. Mumps, whooping cough, scarlet fever, and pneumonia are all epidemic at present in the city, while alternate thawing and freezing—making the streets like glass—is responsible for many accident cases.

### Middlesex Hospital.

PRESENTATION TO NURSE CROSS.

At the conclusion of the general business at the annual court of governors of Middlesex Hospital on Thursday last an interesting ceremony took place, which was witnessed by a large gathering from the medical school. The Board had unanimously decided to present an illuminated address to Nurse Sarah Cross to mark their recognition of her devotion in nursing the wounded at the time of the Jamaica earthquake. The function was performed by Mr. Henry Morris, President of the Royal College of Surgeons, who acted as Chairman in the unavoidable absence of Prince Francis of Teck. Mr. Morris expressed on behalf of the Board the pleasure they experienced at the knowledge that a nurse of the Middlesex Hospital had shown such courage and capacity in so tremendous a crisis. They were all proud of her, and they recognised that it was not only technical training, but personal character and endurance, which had enabled her to accomplish a work which had carried the fame of English nurses as far as the West Indies. It gave the Board great satisfaction to replace all the belongings which Nurse Cross had lost in the earthquake.

Nurse Cross, who on entering the Board-room was accompanied by the matron, Miss Vernet, and the superintendent of the nursing home, Miss Wethered, then, amidst loud cheers from the medical staff and students, advanced and received at the Chairman's hands a framed illuminated address which was in the following terms :—

"This address is presented to Nurse Sarah Cross by the weekly Board of Governors of the Middlesex Hospital in order to mark their high appreciation of her heroic conduct on the occasion of the earthquake at Kingston, Jamaica, on January 14, 1907, when for nearly forty hours consecutively she assisted Dr. Arthur Evans on board the R.M.S. *Port Kingston* in rendering surgical aid to 200 persons injured in the catastrophe. Her fortitude and noble devotion signally redound to the honour of the nursing profession and of British womanhood.

"Cheylesmore,

"Chairman of the weekly Board of Governors."

Nurse Cross, who was again loudly applauded, was so agitated by what must have been a trying ordeal that she was only able to murmur a few words of thanks in reply. The address bears the Middlesex arms, and is ornamented with a red cross at each corner.

### For Inventive Nurses.

The British Red Cross Society are going to hold their Eighth International Conference in London from June 10 to the 14th. Among other exhibits it is hoped that the inventions in connection with the "Empress Marie Féodorovna" Fund will be on show. The Empress gave a sufficient sum of money to award prizes to the value of £2,000 for the best inventions to lessen the sufferings of sick and wounded soldiers, such as the most practicable means of lifting the wounded on the battlefield, the best types of stretchers, and of vehicles for conveying the wounded from the battlefield to the bandaging posts with the greatest possible rapidity and with the least suffering to the wounded, the best installations in railway carriages, on board ship, etc. Those nurses who went through the South African campaign may have some decided ideas as to how improvements may be effected on several of these points. If so, they should apply at once to the Secretary of the British Red Cross Society, 9 Victoria Street, London, S.W., for full particulars, and all persons intending to compete must send in their names and addresses before April 1, 1907.

### The New Canadian Residence for Nurses.

In the magnificent new residence for the nurses of the Hospital for Sick Children at Toronto, which was opened last month, there are several features of interest. Opening out of the demonstration-room is a swimming pool and plunge bath. At the west end of the bath there is a marble platform, and at the north end of the platform a shower bath. In attendance is a gymnastic instructress, who is an expert in swimming. *The Canadian Nurse*, which gives a full description of the building, states that no nurse is allowed to enter the bath alone, and that if an attendant is not in the room she must be accompanied by another nurse. The nurses have the use of a sewing-room, in which two machines are run by electricity; in the library are a thousand volumes of well-selected fiction and general literature; there is also a medical library with 300 volumes; and the corridor contains a "roster" for nurses, invented by Dr. Fisher, of New York, similar to the one in use in the Presbyterian Hospital in that city, and consisting of a board divided into a hundred compartments, each compartment being half an inch wide and three inches long. The object of the "roster" is to enable the individual nurse, by means of a system of pegs, to indicate her whereabouts. If she is on duty in the hospital she places a red peg opposite her name which is inserted in the compartment; if out of the residence a blue peg; if on vacation a white peg; if on outside duty a white and red peg; and if in her room a green peg. There is a gymnasium 14 feet high and 25 by 41 feet wide, and an instructress gives daily instruction of half an hour to different classes, the half-hour being taken out of hospital time. On the top of the building is a roof garden covered with slate work and provided with an awning.

### Confirmation in an Infirmary Ward.

BY A CORRESPONDENT.

Confirmation is always an impressive and solemn service, but it becomes doubly so when conducted in a hospital ward. Some of your readers may be interested to hear of one which took place recently in a Poor-law infirmary. In these institutions patients are often inmates for a long time, frequently in cases of incurable disease till their death.

There were four candidates, women ranging in age from twenty to forty-four, all suffering from some chronic disease. One woman, with a very bad heart, was confirmed in bed.

The Infirmary as yet has no chapel, but an empty ward is used for our services. The improvised altar looked very beautiful against a background of palms on the eventful morning, with its fair white cloth, white tulips and narcissi, and the emblematic cross in the middle. The matron, outside friends, sisters, all the nurses who could be spared from the wards, most of the night nurses, and convalescent patients or those able to be up, were present. Three of the candidates were on lounges quite in front. One of the sisters played the hymns. At 11.30 the Bishop of Wakefield arrived with his chaplain. After the opening hymn and a prayer, the Bishop gave a short explanation of Confirmation; then speaking of the mystery of pain, he told in simple but convincing words of its strengthening and refining effects. The pathos of the three women lying there, with their Confirmation veils, the white surplices of the clergy, the beautiful spring flowers, the earnest, reverent attention on faces lined and haggard with care and disease, and the pink and blue uniforms of the sisters and nurses made a specially impressive and solemn scene, the more so as all knew that for one of the candidates the "Everlasting Kingdom" was not far off.

### Everybody's Opinion.

### A PROBATIONER LOSES HER SIGHT.

"Altere Alterius Auxilio Eget" writes from Tettenhall Court, Wolverhampton, March 2: I see that you are kindly forwarding subscriptions to the probationer at West Ham Infirmary who has injured her eyesight. May I trouble you to send her the enclosed 10s. with my sympathy?

"M. J. T." writes: "I wish to thank, on behalf of my daughter, Probationer Nurse Turner, all kind donors for their sympathy, and also to state that, not having received a satisfactory answer from the West Ham Board of Guardians, I am now in communication with the Local Government Board."

### A LONG-FELT WANT.

"A Puzzled One" writes: Could any nurse advise as to the best shoes to wear on night duty where quietness is absolutely essential? A felt sole meets the requirement to a certain extent, but I find these make the instep ache very much, as there are no heels. Would it be possible to get anywhere a felt silent sole with a heel of some sort fixed on? My bootmaker tells me it is impossible. I should like to know what other nurses do as regards this difficulty?

### POOR-LAW INFIRMARY-TRAINED NURSES.

"Hurt" writes: I shall be pleased if you can find a corner for this letter in your valuable paper. I am quite sure that many infirmary-trained nurses read the Mirror, and they naturally feel the same as I do about the way we are slighted. I should like to know whether we are labouring under a cloud which might very easily be removed by a little explanation. Does "Not Infirmary Trained" mean nurses who have been trained in a large London institution where there are four resident doctors, a large staff of nurses, who are thoroughly trained under sisters who have received their training in large hospitals, and a matron who was London hospital trained? I am extremely anxious to obtain further surgical work, so I wrote to a matron the other day, enclosing stamp for reply. She has simply ignored me. I do not like to think that this is because I am not hospital-trained. Why should there be any difference? Why should a place which is supported voluntarily be any better than one which is kept up by the rates? What reason is there for a probationer to be well educated if she cannot rank with those who may be her inferiors in social standing? My opinion is that the best nurses procurable should be obtained for Poor-law institutions. How can England raise the masses if she provides uneducated women of a common class to tend her sick poor in her public charitable institutions! Just think for a moment what untold good may be done, and what good may be left undone, if the Poor-law trained nurse is to be kept down.

### THE DISTRICT NURSE AND THE DOCTOR.

"Justice" writes: I cannot understand why a medical man should treat "E. W." and "Kentish Nurse" as they say. My experience has been quite the opposite. I have worked for years on district and privately, and always received the greatest consideration and kindness (in case of illness) from the doctors I worked with. The doctor has come at once when sent for, and attended me as any other lady patient, quite free of any charge. I always send for the doctor I like best in the town, and always shall, whether he happens to be the "nurses' doctor" or not. Often when on district if I have looked extra tired, the question, "Tired, nurse?" if answered in the affirmative, has received, "Go home, muffle that bell of yours, and get a good rest." Only a few weeks ago I took a chill. I did not want to trouble the doctor, but as I was unable to work and felt so ill when evening came, sent a note to tell him. He was out at the time, but came as soon as he returned home, visited me every morning until able to resume work.

This is only a sample of the treatment invariably received. One doctor in the South, when I offered to pay him, remarked, " You are one of us." Another, when I said I was sorry to give him so much trouble, made answer, " You know, nurse, I would come to you any time, night or day, and do anything I could for you." Personally, I do not see why medical men should attend nurses free. We are much better paid than many other women who work. Sometimes I think we expect too much. Was the doctor all to blame in the cases mentioned? I should think not.

### IN SEARCH OF A DOCTOR.

" A District Midwife " writes : Practising as a midwife on a London district, I was sent for one evening at 9 P.M. to a case which I was not engaged to attend, but the messenger said that Mrs. —— was intending to engage me only she had not been prepared for the event to come off so soon. I went, put the patient to bed at once, examined her, and found a miscarriage at the fourth month was inevitable. Ante-partum hæmorrhage was going on, and the patient told me, moreover, that she had been losing blood continuously, more or less, for three months. I only wish I had been sent for before, then perhaps with rest and care we might have averted the catastrophe. Of course I said I could not attend the case without a doctor, and sent for one then and there. The patient, not being in very affluent circumstances, could not afford a high fee; but she offered to pay 10s. 6d. I therefore sent for the parish doctor. He told my messenger that he would have come willingly, but the house was just out of his district, so he directed the girl to the house of the parish doctor in whose district we were living. She could not get him to come then, but, after waiting for nearly an hour, he arrived, accompanied by a lady in evening dress, apparently returning from the theatre or a dinner party, and told the poor girl, angrily, " that he could not think of coming without an order from the relieving officer." " What business had the nurse to send him such a message? " By this time it was nearly midnight, far too late to get orders from relieving officers. The hæmorrhage was now temporarily arrested and the pains less strong, so I left my patient for half an hour with a competent attendant, and hurried off to try myself to get a doctor. I tried four—one was out, two refused point blank to come, and the other, from the speaking-tube at his bedside, told me, after hearing and agreeing to the urgency of the case, that he would not get out of bed without the fee being put into his hand. In vain I promised he should have it on arrival at the case, and even went so far as to guarantee it myself. No, he would not get out of bed, however urgent the case might be. So I returned to my patient, and at about 2 A.M. the little dead baby was born. A great deal of post-partum hæmorrhage occurred, causing fainting and collapse. After an hour I examined and found the placenta was retained, undetached from the uterine wall. I controlled the hæmorrhage, and treated the patient for collapse in the usual manner, but, of course, I dare not give ergot, as I could not empty the uterus. I expressed several very large clots. Finally, soon after 4 A.M. I left the patient again for a short time and fetched one of my own nurses to assist me. Then at 7 A.M. I sent this nurse to try once more to get a doctor. She tried three or four, but without success. At eight o'clock I went again myself to see one of these, and begged him at least to give me instructions what to do. All he told me I knew already—namely, that at 10 A.M. I could get an order from the relieving officer which would oblige the parish doctor to come. I could not leave my patient again, as she was in a most critical condition, therefore I sent one of my nurses on this errand, and finally about midday the doctor actually did arrive. He did exactly as I had already done (with this exception, that he did not disinfect his hands at all), examined the patient, and gave a verdict of retained placenta, and recommended her removal to the infirmary to be curetted. This was carried out at four o'clock the same day, and I am glad to say that my patient is now going on well. Decidedly, no one can deny that my experience in this neighbourhood of the doctors is distinctly unfortunate, as they evidently believe that a night's rest in bed is of more value than a patient's life.

### Appointments.

Alnwick Infirmary.—Miss M. A. Rutledge has been appointed matron-nurse. She was trained at the Nurses' Home and Training School, Frederick Street, Belfast, and has since been working under the Alnwick District Nursing Association.

Children's Hospital, Bradford.—Miss M. Gallaway Roberts has been appointed night charge nurse. She was trained at the Royal Southern Hospital, Liverpool, and has since done private nursing.

Edmonton Urban District Council.—Miss Florence Tettenham has been appointed lady inspector and health visitor in the Public Health Department. She holds the certificates of the Sanitary Inspectors' Association and of the Central Midwives Board; also that of the National Health Society.

Cossham Memorial Hospital, Bristol.—Miss Marian Maun has been appointed matron. She was trained at the Preston Royal Infirmary, and has since been charge nurse at the Park Hospital, Lewisham; night sister at the Oldham General Infirmary; sister at St. Mary's (Islington) Infirmary, Highgate Hill, London, N.; and for the last two years assistant matron at the General Infirmary, Gloucester.

General Infirmary, Gloucester.—Miss Eleanor Edwards has been appointed theatre sister. She was trained at the City Fever Hospital, Coventry, and afterwards for four years at the Royal Infirmary, Sheffield.

Hertford Memorial Hospital, Leicester.—Miss Margaret J. Edwards has been appointed matron. She was trained at the Sheffield Royal Infirmary, and has since been staff nurse at the Borough Hospital, St. Helens; sister at the Infectious Diseases Hospital, Cambridge; sister at the City Hospital, Nottingham; and sister-in-charge of enteric wards at the Sanatorium, Hull.

Hospital for Epilepsy and Paralysis, Maida Vale, London.—Miss R. S. Weston has been appointed matron. She was trained at the London Hospital, Whitechapel, and has since been sister at the Royal Hospital for Incurables, Putney; out-patient sister at the Royal Ophthalmic Hospital City Road, London; assistant matron at the City of London Hospital for Diseases of the Chest, Victoria Park, E.; and matron of the Central London Ophthalmic Hospital, Gray's Inn Road, London.

Jessop Hospital for Women, Sheffield.—Miss Edith Amelia Corbett has been appointed staff nurse. She was trained at Wolverhampton Union Infirmary, and holds the certificate of the Central Midwives Board.

Manchester Workhouse Infirmary, Crumpsall.—Miss Ellen E. Powell, Miss Fanny Spriggs, Miss Jane Coward, Miss Isabella G. Mollison, Miss Lily Gray, and Miss Kate Joyce have been appointed ward sisters. They were trained at Crumpsall Infirmary.

Reading Infirmary.—Miss Martha Louisa Mancer has been appointed superintendent nurse. She was trained at Steyning Union, where she was afterwards charge nurse. She has since been superintendent nurse at Pontypridd, Christchurch, and Wallingford Unions.

Tetbury Cottage Hospital.—Miss Alice E. Keeble has been appointed matron. She was trained at the Royal Albert Edward Infirmary and Dispensary, Wigan, where she has since held various posts.

Throne Hospital, Belfast.—Miss Helena Elliott has been appointed charge nurse. She was trained at County Antrim Infirmary, Lisburn, where she afterwards became charge nurse. She has since been charge nurse at the Samaritan Hospital, Belfast; charge nurse at County Louth Infirmary; and charge nurse at the Unicn Infirmary, Antrim.

TANFIELD ISOLATION HOSPITAL, TANTOBIE, NEWCASTLE.—Miss C. Clarke has been appointed charge nurse. She was trained at St. Mary (Islington) Infirmary, London, and has been on the private staff of the Norfolk and Norwich Hospital and sister at Shirley Warren Infirmary, Southampton.

WORTLEY UNION, SHEFFIELD.—Miss Gertrude M. Blackburn has been appointed superintendent nurse. She was trained at the Bagthorpe Infirmary, Nottingham. She has also been nurse at the Ilkeston Accident Hospital, assistant nurse at the Grimsby Union, and charge nurse at the North Evington Infirmary, Leicester. She holds the certificate of the Central Midwives Board.

### Queen Victoria's Jubilee Institute for Nurses.

MISS ADDENBROOKE has been transferred to Winsham, from Northampton; Miss C. M. N. Bell to Wetherby, from Hertford; Miss E. M. Buller to Norwich, from Oxford; Miss Florence Green to Huddersfield, from Gloucester; Miss Dora M. Laycock to Withnell, from Blackburn; Miss Alice Pennington to Arnold, from Leighton Buzzard; and Miss Janet Watson to Ashton-under-Lyne, from South Tottenham. Miss Eva Sissons has been appointed to Guildford; and Miss Lydia D. Nesbitt temporarily to Lancaster.

### The Nurses' Bookshelf.

LESSONS ON MASSAGE. By MARGARET D. PALMER. (Baillière, Tindall, and Cox. Third edition. Pp. xvi.+272. Price 7s. 6d. net.)

THIS book, the authoress of which was formerly masseuse and manager of the massage department of the London Hospital, has been enlarged, certain sections of it being entirely rewritten. The increasing amount of literature on this subject is, no doubt, owing to the fact that the medical profession is now making more use of massage in the treatment of various surgical and medical affections, and we can strongly recommend this book as a reliable guide to students of the subject. The different methods of using massage are described in a manner which can be easily followed, and shows that the writer has a thorough grasp of the subject. The anatomical plates and letterpress add to the interest and value of the book; by referring to them the reader can intelligently follow the details of treatment. On page 88 there is a misprint: flexor longus pollicis is called "the longest pollicis." The book is beautifully printed, and the illustrations are exceptionally well done.

### A COMPETITION FOR CHRISTMAS, 1907.

THE success of our experiment last year in inviting nurses to write our Christmas Supplement induces us to afford them the opportunity next year of contributing to our columns a special short story bearing upon hospital, infirmary, mental asylum, or district or private nursing life, not necessarily consisting of actual facts, but, if possible, founded upon them. The length of the story, which must, of course, be interesting and probable from first to last, should be about 5,000 words, and, other things being equal, preference will be given to the contribution which is accompanied by two or three good photographs or drawings, for purposes of illustration. If possible it should deal with the Christmas season. The sum offered for the story is £5 5s., and the competition is open to nurses in all parts of the world. In order to stimulate effort, we shall send a cheque for £2 2s. to the author of the story which we consider second best, reserving the right to publish it. With the view of enabling our readers in the Far East and other distant quarters of the globe ample time to forward MS., contributions will be received up to June 30, 1907; but we shall be glad to have them as soon as convenient. They should be addressed to the Editor, Nursing Section of THE HOSPITAL, and marked outside "Christmas Competition."

### Presentations.

BOROUGH HOSPITAL, BOLTON.—Miss Marian Stevenson, who has relinquished the post of matron of the Borough Hospital, Bolton, to take up duty in Huddersfield, has been presented by the nursing and domestic staff with a morocco leather dressing-case.

WHITSTABLE DISTRICT NURSING ASSOCIATION.—At the last general meeting of the committee of the Whitstable District Nursing Association, Nurse May, who has left on account of failing health, was presented with a small bureau, an album containing a letter of farewell, and the names of the 453 subscribers, and a cheque for £46 8s. 4d.

### TRAVEL NOTES AND QUERIES.

BY OUR TRAVEL CORRESPONDENT.

BRITTANY IN EARLY SPRING (Berrie).—Let me hear at once how much money you wish to spend on your holiday, including the journey; also whether the journey is to be made within the fortnight or outside it. The roads are beautiful where you wish to go, and there are places of much interest. As to temperature, it is about the same as England at that time, but there is more sun. I cannot map out your tour till I know what you can spend, but shall have pleasure in helping you when I have particulars. I should like to know also whether you prefer coast or inland scenery.

HOLLAND IN EARLY SPRING (Penstowe).—You give no pseudonym, but hope you will recognise this. I do not think you can manage a fortnight on less than £12. Ask for tickets *via* Harwich, Rotterdam, Amsterdam, Hoorn, Alkmaar, Leyden, and The Hook. Cost about £2 second class return to England. Spend two nights at Rotterdam—Hotel St. Lucas, 327 Hoogstraat, or Grand Hotel, Coomans, 12 Hoofdsteg. Whilst there visit Dordrecht, Delft, and Gouda, all easy excursions. On the third day go to Amsterdam—Pension Bellevue in the Sarphatistraat, or Hotel Haas in the Papenbrugsteeg. Stay there five nights, visiting the island of Marken, Haarlem, and Zaandam. On the eighth go to Hoorn—Hotel Central. If the steamers are running so early go by one; it is more interesting. Stay two nights, visiting Enkhuizen, etc. On the tenth day go to Alkmaar—Hotel Toelast or Hotel de Burg. Stay one night. Leave early; reach The Hague in the evening—Hotel du Passage or Hotel d'Angleterre, 22 Eerste Wagenstraat. Stay three days, visiting Leyden. To make both ends meet, take your breakfast and dinner in the hotels, but your lunch you must arrange yourselves. Ask always for rooms on third floor.

CHEAPEST ROUTE TO VENICE (Countrywoman).—Thanks for P.O.O. I am glad our last arrangements turned out so well; we must try to be equally successful this time, but it is a long journey to take for so short a holiday. Your best route is to go and return *via* Dieppe, the St. Gothard, and Milan, £8 5s. 9d. second class return. The Mont Cenis way is dearer and not nearly so beautiful. Stay one night at Milan, go to Pension Viviani in the Via Gabria Casati, or Hôtel Passarella in the Via Passarella. Your room will be about 2½ lire, and dinner 3½, early breakfast 1 lire. At Venice go to Pension Gregori Palazzo, Barbarigo Grand Canal, or Pension Lewald, 743 Fondamento S. Vio, or Pension Bril-Da-Run. All these will charge 6 or 7 francs, according to the size and position of your room. If you need help as to how to spend your time to advantage, write me again.

RULES IN REGARD TO CORRESPONDENCE FOR THIS SECTION.—All questioners must use a pseudonym for publication, but the communication must also bear the writer's own name and address as well, which will be regarded as confidential. All such communications to be addressed "Travel Correspondent, 28 Southampton Street, Strand." No charge will be made for inserting and answering questions in the inquiry column, and all will be answered in rotation as space permits. If an answer by letter is required, a stamped and addressed envelope must be enclosed, together with 2s. 6d., which fee will be devoted to the objects of "The Hospital" Convalescent Fund. Ten days must be allowed before an answer can be published.

### Notes and Queries.

**REGULATIONS.**

**The Editor is always willing to answer in this column, withou any fee, all reasonable questions, as soon as possible.**

**But the following rules must be carefully observed.**

**1. Every communication must be accompanied by the name and address of the writer.**

**2. The question must always bear upon nursing, directly or indirectly.**

**If an answer is required by letter a fee of half-a-crown must be enclosed with the note containing the inquiry.**

*District Nurses to Population.*

(224) Can you inform me what population is considered the correct average for one district nurse to undertake? I am Secretary to a Village Nurses' Association, where one nurse undertakes midwifery and general nursing. The population is about 2,000 and it is a large, scattered district with hamlets four miles out of the village which have to be undertaken. The work is at times very heavy, and I should be glad to know the experience of any district nurses similarly situated.—*Secretary.*

We invite our readers to reply, but it is manifest that one district nurse to a population of 2,000 residing in a large and scattered district, with hamlets four miles apart, is quite inadequate. If one hospital bed is required for each 1,000 of the population, there should certainly be at least one nurse to every 1,000 inhabitants in scattered country districts.

*New York.*

(225) My friend and I are anxious to start private nursing in New York. We are fully qualified. Please give me all information?—*Gloucester.*

We cannot advise you too strongly not to take such a step. America is well supplied with nurses, most of whom have gone through a very thorough training. You have no chance of success whatever. Rent and living are very expensive.

*Nursing in India.*

(226) Is there a nursing home in Calcutta especially for midwives or maternity nurses? What is the address of the Colonial Nursing Association?—*Wales.*

We know of no such institution. You might get information useful to you if you write to the Secretary, The Indian Nursing Association, Simla, India. The address of the Colonial Nursing Association is Imperial Institute, S.W.

*Nurse on Board Ship.*

(227) I am anxious to obtain a post as nurse on board a ship. Where should I apply?—*Stamford.*

Two shipping companies employ nurses on board ship—the Royal Mail Packet Co., Southampton, and the Booth Steamship Co., Liverpool; but vacancies are exceedingly rare.

*School Nurse.*

(228) I am a trained nurse; to whom should I apply for a post as nurse in a boys' or girls' school (public or private)?—*Southampton.*

Advertise in a scholastic or daily paper.

*Probationer.*

(229) I wish to train as a nurse. Can you tell me my best method of procedure, or in which direction must I seek information? Should I receive a salary during my probationership, and if so, how much?—*Jessica.*

Consult "How to Become a Nurse," price 2s. 4d. post free, The Scientific Press, 28 and 29 Southampton Street, Strand, London, W.C. When making an application, write to the matron in your own handwriting.

*Nursing Home.*

(230) I should like to know if there is any home either in Edinburgh or Glasgow which one could enter without a premium. Does a masseuse require general nursing training?—*Edinburgh.*

The Royal Scottish Nursing Institution, Queen Street, Edinburgh; Training Home for Nurses, 250 Renfrew Street, Glasgow. A masseuse need not train as a nurse, but it is desirable that she should.

**Handbooks for Nurses.**

| | Post Free. |
|---|---|
| "A Handbook for Nurses." (Dr. J. K. Watson) ... | 5s. 4d. |
| "How to Become a Nurse." (Sir Henry Burdett) ... | 2s. 4d. |
| "Nurses' Pronouncing Dictionary of Medical Terms" | 2s. 0d. |
| "Art of Massage." (Creighton Hale) ... ... ... | 6s. 0d. |
| "Surgical Bandaging and Dressings." (Johnson Smith.) ... ... ... ... | 2s. 0d. |
| "Surgical Instruments and Appliances Used in Operations," illustrated. (Harold Burrows, F.R.C.S.) | 1s. 8d. |
| "Hints on Tropical Fevers." (Sister Pollard.) ... | 1s. 8d. |

Of all booksellers or of The Scientific Press, Limited, 28 & 29 Southampton Street, Strand, London, W.C.

### For Reading to the Sick.

### THE SYMPATHY OF CHRIST.

If I could only surely know
That all the things that tire me so
  Were noticed by my Lord—
The pang that cuts me like a knife,
The lesser pains of daily strife—
  What peace it would afford!

It seems to me, if sure of this,
Blent with each ill would come such bliss
  That I might covet pain,
And deem whatever brought to me
The loving thought of Deity
And sense of Christ's sweet sympathy,
  Not loss, but richest gain.

Dear Lord, my heart shall no more doubt
That Thou dost compass me about
  With sympathy divine:
The love for me once crucified
Is not the love to leave my side,
But waiteth ever to divide
  Each smallest care of mine.

*Anon. (American).*

One of the loveliest mysteries of the incarnate life was the sympathy of our blessed Lord. He not only entered into the sorrows and sufferings of mankind, but really suffered along with them. "He bare our sicknesses." When we speak of sympathy and compassion, this is what the words literally mean—suffering with others—not only feeling for them, but suffering with them. And such was the sympathy of Jesus Christ—the only perfect sympathy that has ever been—the prerogative of His perfect humanity. His was that wholly unselfish sympathy with others which permitted and enabled Him not only to regard their sufferings and sorrows with a pitying eye, but to take them into His own heart. And still He is "touched with the feeling of our infirmities" (the word in the Greek is "sympathise"). In what manner or under what conditions this can be, in His now glorified state, we cannot tell, but the truth remains though we may not understand it; and every sufferer, whether in acute pain or simple weakness (for the word "infirmities" is really "want of strength"), such sufferer may reckon upon the true and perfect sympathy of the Son of God, and may realise through that confidence a closer and a sweeter fellowship with Him.—*Bishop of Lichfield.*

The belief of a Man who suffers with us becomes, then, not a theory, but a fact: one which we may recognise, though we may feel very little of the joy or satisfaction which it might seem to be full of. For all deep truths must be found out, I think, slowly. They lie beneath all experiences of pleasure or pain. We are to grow with them, and in due time they will work upon us, and mould us after their own likeness. May God be with you, and make you know more of Himself than any words can tell.—*Dean Goulburn.*

## Figures and Tables

**Figure f1:**